# Detecting differential gene expression in blastocysts following pronuclear transfer

**DOI:** 10.1186/s13104-017-2421-3

**Published:** 2017-02-15

**Authors:** Edward H. Morrow, Fiona C. Ingleby

**Affiliations:** Evolution, Behaviour and Environment Group, School of Life Sciences, University of Sussex, John Maynard Smith Building, Brighton, BN1 9QG UK

## Abstract

Nuclear transfer techniques (a.k.a. mitochondrial replacement therapies) are currently under development to provide a route to eliminating particular instances of mitochondrial disease from the germline. Before these kinds of techniques are implemented clinically it is of primary concern that their safety and efficacy is established. In a recent paper, Hyslop et al. (Nature 534:383–386, 2016. doi:10.1038/nature18303) utilized a specific version of pronuclear transfer to investigate the consequences for gene expression in the developing embryo, which may indicate whether or not developmental pathways have been perturbed. However, the study was only able to include a small number of blastocysts within each treatment group, although a larger number of single cell expression profiles from each blastocyst were acquired. Using simulated datasets we show that the size and experimental design of this study cannot provide conclusive evidence that expression profiles of manipulated or control samples are indistinguishable from one another due to low power. These simulations also illustrate why visual inspections of principle component analyses used in the study cannot replace statistical modeling of treatment effects.

**Electronic supplementary material:**

The online version of this article (doi:10.1186/s13104-017-2421-3) contains supplementary material, which is available to authorized users.

## Background

Two main methods of mitochondrial replacement—pronuclear transfer (PNT) and maternal spindle transfer (MST)—are currently under development as potential germline therapies for eliminating some forms of mitochondrial disease. Hyslop et al. [1] examined the consequences for early stage embryos following an ‘early’ version of PNT (termed ePNT), where zygotes had completed meiosis but not yet undergone mitosis. Gene expression profiles were obtained from single cell samples of blastocysts created using four different main methods: ePNT of oocytes from two different unrelated women (heterologous, n = 9), unmanipulated controls (n = 3), and two types of procedural controls—ePNT of oocytes from the same donor (autologous, n = 1), and ePNT of oocytes from two related sisters (homologous, n = 1). Including autologous and heterologous controls potentially enables the authors to disentangle the effects of the ePNT procedure itself from any effects that may arise from switching the nuclear genomes between different mitochondrial genetic backgrounds. This mitonuclear mismatching is a potential safety concern for the clinical implementation of any of the various versions of mitochondrial replacement therapy [2, 3]. RNAseq data from blastocyst-derived single cells were explored via principle component analysis (PCA), t-distributed stochastic neighbour embedding, and unsupervised hierarchical clustering. On the basis of these exploratory analyses, the authors concluded that gene expression levels were indistinguishable between control and ePNT blastocysts.

However, there a number of shortcomings to the analytical approaches undertaken. First, the power to detect differences between treatment groups is low due to the small number of biologically independent samples, which is at the level of blastocyst and not single cell sample. For instance, a test of the mitonuclear mismatching hypothesis would compare nine heterologous versus a maximum of two autologous/homologous blastocysts. Second, no statistical modeling of treatment effects was conducted, which obviously precludes the possibility of making any conclusions about whether or not there are statistical differences overall, or between specific treatment groups. The analyses only extend as far as plotting the results of a principal components analysis (PCA), which is a variance-orientated dimension reduction technique that can be useful for preliminary visualization of data.

We investigated these issues using simulated datasets and subsequent power analysis and principal components analysis, and conclude that based on the number of samples included and the magnitude of effect sizes that might reasonably be expected to be present, the study is unable to provide clear evidence that the manipulated samples are indistinguishable from controls.

## Methods

The power to detect differential gene expression between treatments was examined via simulation, where simulated datasets based on the experimental design used here were analysed for differences between treatments using a mixed effects linear model. In order to resemble a transcriptomic analysis of differential gene expression, simulations were ran in batches of 100 (i.e. analogous to analysing 100 genes) and the power was calculated from each batch as the percentage of significant tests. These batches were repeated to produce 100 power estimates from simulated data. Two sets of simulations were ran: Set 1 tested a range of effect sizes, and Set 2 tested a range of sample sizes. All analyses used R v3.2.1 and the ‘lmer’ mixed modelling function in the ‘lme4’ package [4]. Methods are described below, and annotated R code that also generates two plots is provided in Additional file 1.

Each simulated dataset was set up by initially specifying a small effect size for differences in gene expression between cell types, variance estimates (based on the median gene expression variance calculated from supplementary data in Hyslop et al. [1]) for both the overall error variance and the variance between blastocysts, and the effect size for treatment. In Set 1, the effect size for treatment was tested for all values between 1 and 10, whereas in Set 2, the effect size for treatment was fixed at 2. The effect sizes as shown are unstandardized, but when standardized using the error variance specified in the models, i.e. with a standard deviation = 10, an effect size of 1 is approximately d = 0.1 (very small; see Cohen [5] for more details on d, which provides an indication of standardized differences in mean values between groups) and an effect size of 10 is approximately d = 1 (very large).

Next, the experimental design for each simulated dataset was set up as a balanced design, based on the numbers of samples in Hyslop et al. [1] (although the actual study is unbalanced). The first set of simulations used 8 blastocysts with 4 samples from each blastocyst (by comparison, Hyslop et al. [1] successfully sequenced RNA from 10 grade A–D blastocysts, with between 1 and 11 samples sequenced from each). In the simulated data, samples were split across a fully factorial design between four different cell types (primitive endoderm, epiblast, trophectoderm and ambiguous) and four different treatments (control, autologous, homologous and heterologous). These factors represent the four cell types and four treatments in Hyslop et al. [1], although samples were unbalanced across these factors. As in the study, all samples from the same blastocyst were under the same treatment. Set 2 of simulations varied the total number of blastocysts, but scaled the experiment to have the same fully factorial design as the Set 1 simulations. Note that simulations were run with an unbalanced design that more closely matched the variable levels of replication in Hyslop et al. [1], and very similar power estimates were obtained.

To simulate the data, gene expression values were generated as the sum of cell type and treatment effects (calculated using the effect sizes), as well as blastocyst and error variance estimated from the data in Hyslop et al. [1]. Note that the study used multiple controls within these four treatments, and so differences in gene expression might only be expected to occur between some of the four levels of treatment, rather than between all. However, for completeness the simulations build in differences between all four treatment groups. If anything, this generates more defined differential gene expression between groups than might be expected in the real data. The data was analysed in a mixed linear model as follows:

$$Y \sim T + C + B + \varepsilon$$

where Y is the simulated expression data, T and C are 4-level fixed factors representing treatment and cell type, respectively, and B is a random factor representing blastocyst ID. P values for the treatment effect were obtained by model simplification via the ‘anova’ model comparison in R [6]. This simulation process was re-run separately for treatment effect sizes 1–10 (assuming 8 blastocysts; Set 1), and then separately for 48, 96, 144, 192 and 240 blastocysts (assuming a treatment effect size of 2; Set 2). Results are shown as the mean of 100 power estimates for each effect size (Set 1) and the mean of 100 power estimates for each blastocyst sample size (Set 2), with 95% confidence intervals.

In the original manuscript, linear models such as those simulated here were not used to determine significance of differential expression between treatments. Instead, a principal components analysis (PCA) of the gene expression data was carried out, and the resulting scores of each sample along principal component vectors were plotted in order to visualize and distinguish between samples based on treatment group. We therefore followed this approach with a final set of simulations, where we generated gene expression values for a multivariate dataset of 12,000 genes (the number of genes analyzed with PCA in Hyslop et al. [1]). This data simulation was carried out exactly as previously, with only one alteration to the code: from one gene to the next, we randomized the order of the treatment levels. This is an important consideration for a multivariate analysis, as without this step, we would be making the unrealistic assumption that every gene differed in exactly the same direction between treatments. We ran these simulations in a variety of scenarios representing different effect sizes, and different percentages of genes that were significantly differentially expressed between treatments. We ran PCA on each simulated dataset, and plotted the samples along PC1 and PC2 to visualize any clustering patterns. While it is difficult to generalize the results of repeated runs of this simulation (due to the nature of PCA, different PC vectors arise for each different dataset), we provide typical examples of plots under different simulation conditions and provide the R code for running them (see Additional file 1).

## Results

**Fig. 1:**
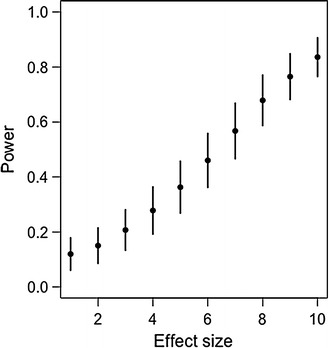
Simulated power based on unstandardized effect sizes. Results shown are the mean power estimate (±95% confidence intervals) for 100 simulations at each effect size (ranging from 1 to 10). Effect size simulations use a similar experimental design and size as the experiment described in Hyslop et al. [1]

**Fig. 2:**
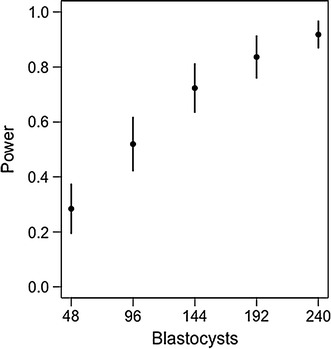
Simulated power based on datasets with varying number of blastocysts. Results shown are the mean power estimate (±95% confidence intervals) for 100 simulations for each number of blastocysts (ranging from 8 to 240). Blastocyst number simulations use a similar experimental design as described in Hyslop et al. [1], but scale the design to increase the number of blastocyst samples

The simulated analysis of differential expression between treatments, based on this experimental design, clearly demonstrates that reasonable statistical power to detect treatment effects would only be possible if: (1) effect sizes were unusually strong (Fig. 1); or (2) a far higher number of blastocysts were sequenced (Fig. 2).

**Fig. 3:**
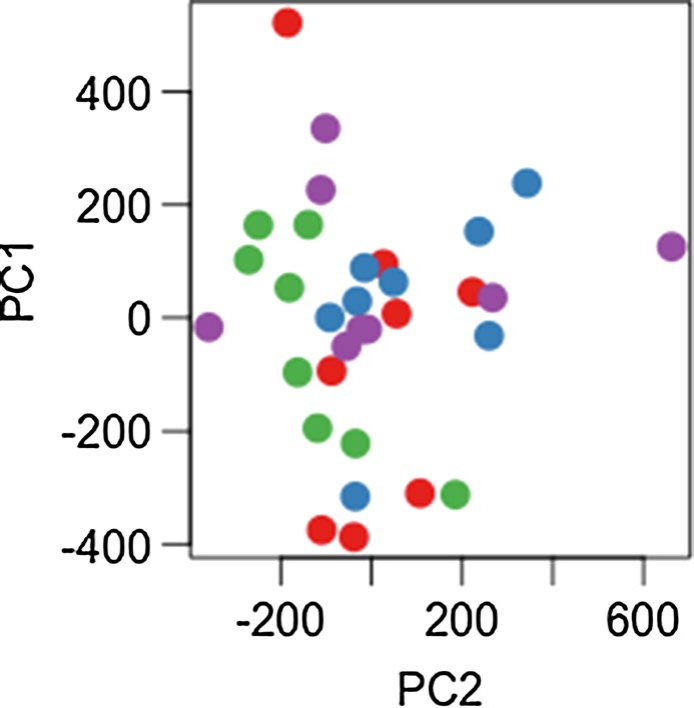
Principal components 1 and 2 plotted according to treatment group, where Cohen’s d = 0.1 for 10% of genes in a simulated multivariate dataset of 12,000 genes. *Different coloured points* represent four different treatment groups

**Fig. 4:**
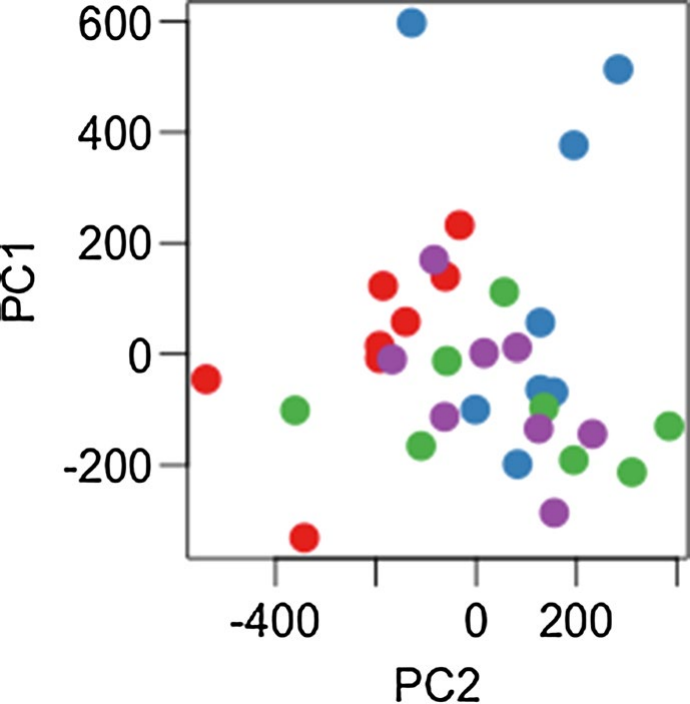
Principal components 1 and 2 plotted according to treatment group, where Cohen’s d = 0.5 for 1% of genes in a simulated multivariate dataset of 12,000 genes. *Different coloured points* represent four different treatment groups

Furthermore, PCA plots of simulated multivariate datasets fail to reveal any clear clustering of samples based on treatment group, even with significant differential expression of genes generated in the simulated data. We show this for scenarios where 10% of all genes (1200 of 12,000) have a low effect size (Cohen’s d = 0.1) for differential expression between treatments (Fig. 3), and where 1% of all genes (120 of 12,000) have a moderately strong effect size (d = 0.5; Fig. 4). We have also included an expanded set of scenarios in Additional file 2. These examples make it clear that even when significant gene expression differences exist between treatments, the approach of plotting principal components to visualize clustering can lead to misleading conclusions about differential gene expression. Such plots can be very useful for visualizing data, but should absolutely be coupled with thorough analysis of the data (as with our linear model simulations) to determine if there are significant differences between groups. These plots may indicate whether or not there is overlap in the distribution of gene expression of different sample types, but as demonstrated in Figs. 3 and 4, significant differences in average gene expression between groups can easily be obscured.

## Conclusions

On the basis of the low power and the descriptive nature of the methods employed by Hyslop et al. [1], the conclusion that blastocysts created via ePNT versus controls, or between the different ePNT treatments are indistinguishable from one another is premature until sufficient data is available to carry out statistical modelling. Any study that aims to establish whether manipulations to embryos following MR can cause significant changes in gene expression should employ proper statistical procedures for detecting possible effects, rather than rely on data visualization from PCA or other variance reduction techniques, as these methods can be misleading and miss real differences. That differences between cell types derived from blastocysts were apparent in PCA plots in Hyslop et al. [1] does not negate the possibility that differences between treatments may also exist. Sampling multiple cell lines from within single blastocysts cannot replace true biological replication. In situations where sample sizes within treatment groups are logistically constrained to small absolute numbers, resampling methods may be a useful approach to improve statistical power [7].

## Additional files

**Additional file 1.** Annotated R code. This script contains the R code for all the simulations described in the manuscript, along with detailed annotations that explain each step of the analyses.

**Additional file 2.** PCA plots for expanded set of simulations. These plots follow from the PCA simulations and Figs. 3 and 4 in the main text for an expanded set of simulations for a range of effect sizes and percentages of differentially expressed genes. Each panel shows principal components 1 and 2 plotted according to treatment group, with different coloured points representing the four different treatment groups. Columns left to right represent effect sizes 1 to 4, and rows top to bottom represent 1, 2, 5 and 10% of 12,000 genes with differential expression between treatments. Note that all datasets are simulated with some extent of significant differential gene expression between treatments, but visual evidence of clustering is only clear when 5% of genes have an effect size of 4, or 10% of genes have an effect size over 3.

## Abbreviations

PNT: pronuclear transfer
MST: maternal spindle transfer
ePNT: early pronuclear transfer
RNAseq: RNA sequencing
PCA: principle component analysis
