# Supplementary figures and images for: Detecting differential gene expression in blastocysts following pronuclear transfer
Source: BMC Res Notes. 2017 Feb 15;10:97. doi: 10.1186/s13104-017-2421-3 (PMC5311846; doi:10.1186/s13104-017-2421-3)

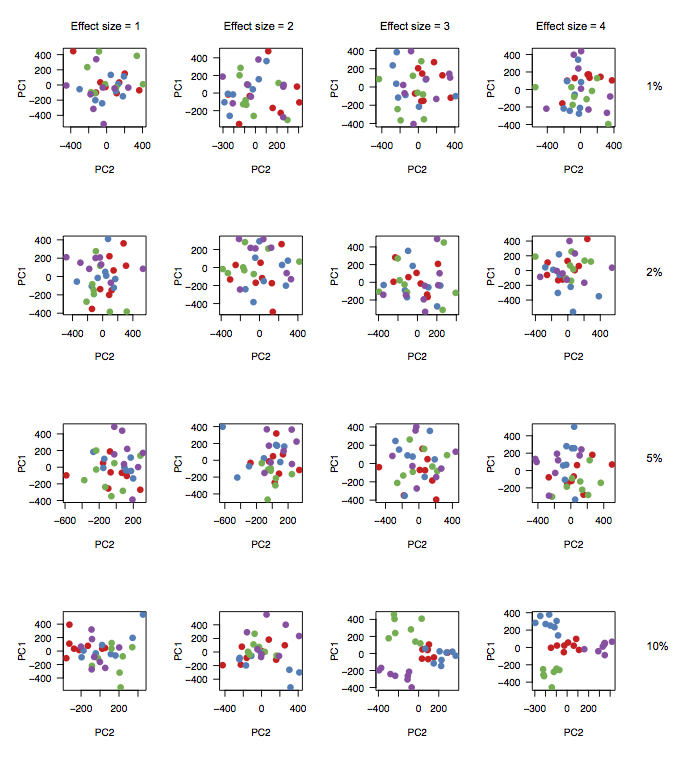

Supplement: Supplementary file 2 — Additional file 2. PCA plots for expanded set of simulations. These plots follow from the PCA simulations and Figs. 3 and 4 in the main text for an expanded set of simulations for a range of effect sizes and percentages of differentially expressed genes. Each panel shows principal components 1 and 2 plotted according to treatment group, with different coloured points representing the four different treatment groups. Columns left to right represent effect sizes 1 to 4, and rows top to bottom represent 1, 2, 5 and 10% of 12,000 genes with differential expression between treatments. Note that all datasets are simulated with some extent of significant differential gene expression between treatments, but visual evidence of clustering is only clear when 5% of genes have an effect size of 4, or 10% of genes have an effect size over 3. [file 13104_2017_2421_MOESM2_ESM.tiff]
